# Supplementary material for: Microclimate of Grape Bunch and Sunburn of White Grape Berries: Effect on Wine Quality
Source: Foods. 2023 Feb 1;12(3):621. doi: 10.3390/foods12030621 (PMC9914167; doi:10.3390/foods12030621)
Supplement: Supplementary file 1 [file foods-12-00621-s001.zip › foods-2166614-supplementary.pdf]

Microclimate of Grape Bunch and Sunburn of White Grape Berries: Effect on Wine Quality

Laura Rustioni [1,*], Alessio Altomare [2], Gvantsa Shanshiashvili [2], Fabio Greco [1], Riccardo Buccolieri [1], Ileana Blanco [1], Gabriele Cola [3] and Daniela Fracassetti [2,*]

[1] Department of Biological and Environmental Sciences and Technologies, University of Salento, Via Provinciale Monteroni, 73100 Lecce, Italy

[2] Department of Food, Environmental and Nutritional Sciences (DeFENS), Università degli Studi di Milano, Via G. Celoria 2, 20133 Milan, Italy

[3] Department of Agricultural and Environmental Sciences (DISAA), Università degli Studi di Milano, Via G. Celoria 2, 20133 Milan, Italy

*** Correspondence: laura.rustioni@unisalento.it (L.R.); daniela.fracassetti@unimi.it (D.F.)**

Table of contents

	Page
**Figure S1:** Canopy appearance the 13 July 2021. On the left: not defoliated row; on the right: total leaf removal in the bunch zone to induce sunburn symptoms.	3
**Figure S2**: Example of berry classification based on the sunburn symptoms: (a) no symptomatic berries, (b) amber colored berries, (c) severely damaged berries, (d) completely dry berries.	4
**Figure S3:** Impact of the canopy management on the berry quality. On the left, an example of a bunch obtained in the leaf removed row; on the right, an example of a bunch collected in the not defoliated condition.	5

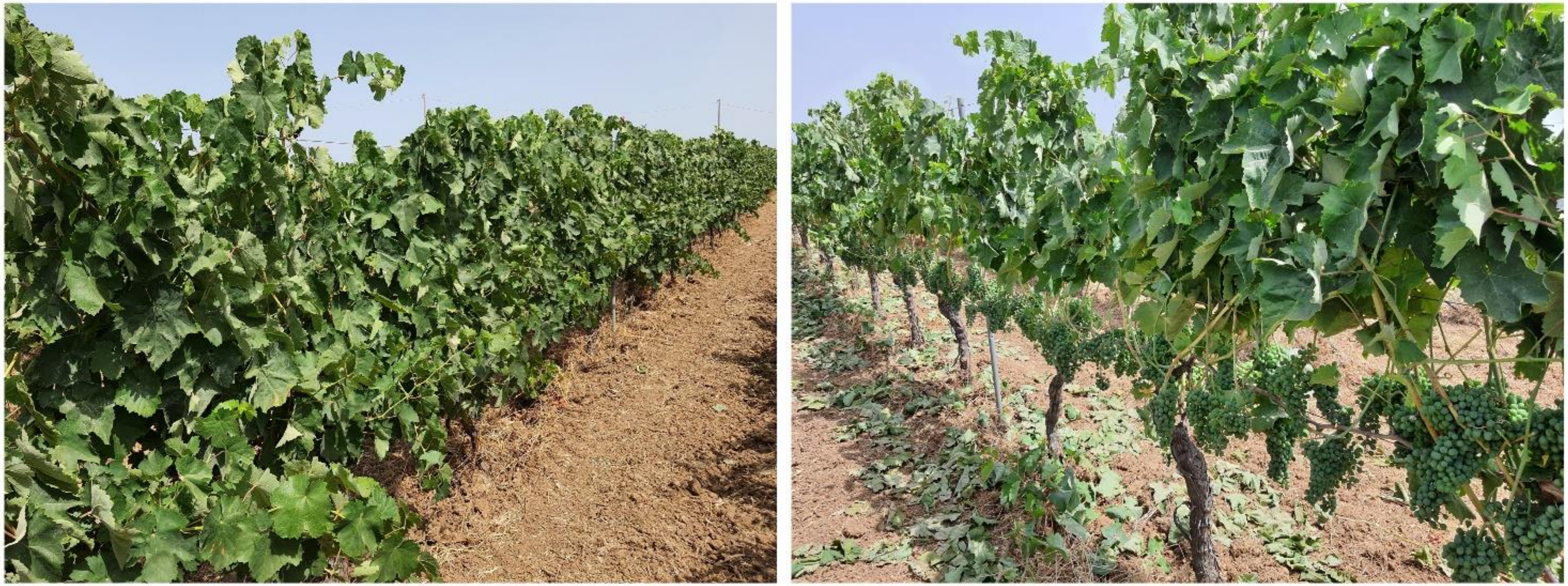

Figure S1. Canopy appearance the 13 July 2021. On the left: not defoliated row; on the right: total leaf removal in the bunch zone to induce sunburn symptoms.

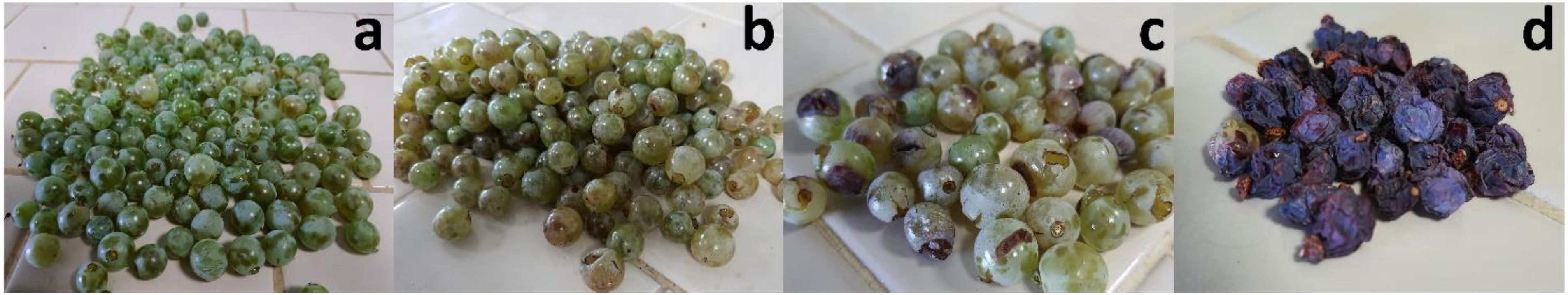

Figure S2. Example of berry classification based on the sunburn symptoms. (a) asymptomatic berries, (b) amber colored berries, (c) severely damaged berries, (d) completely dry berries.

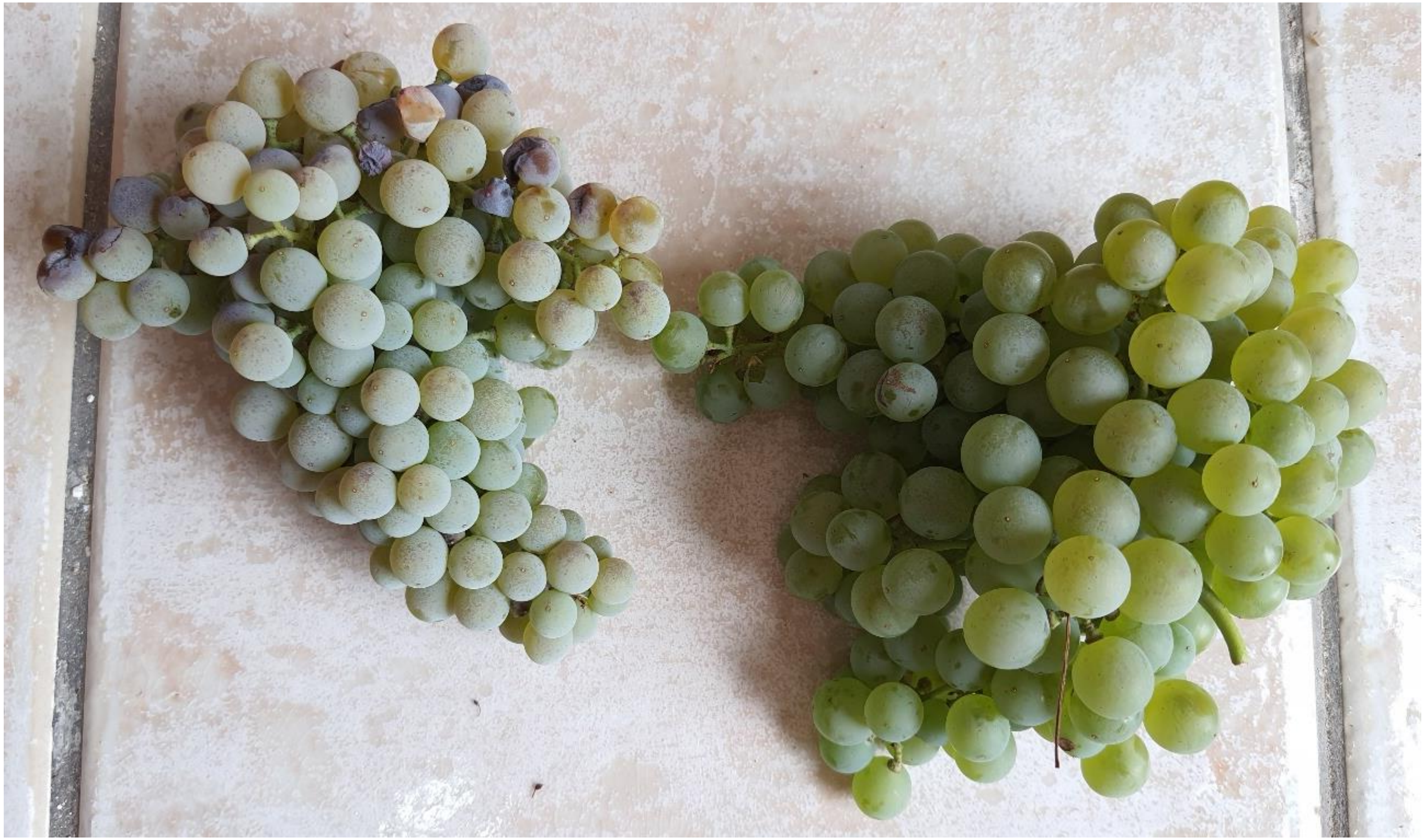

Figure S3. Impact of the canopy management on the berry quality. On the left, an example of a bunch obtained in the leaf removed row; on the right, an example of a bunch collected in the not defoliated condition.
